# Sarcopenia and preserved bone mineral density in paediatric survivors of high‐risk neuroblastoma with growth failure

**DOI:** 10.1002/jcsm.12734

**Published:** 2021-06-29

**Authors:** Michelle Guo, Babette S. Zemel, Colin P. Hawkes, Jin Long, Andrea Kelly, Mary B. Leonard, Diego Jaramillo, Sogol Mostoufi‐Moab

**Affiliations:** ^1^ Department of Pediatrics, The Children's Hospital of Philadelphia, Perelman School of Medicine University of Pennsylvania Philadelphia PA USA; ^2^ Center for Artificial Intelligence in Medicine and Imaging Stanford University Stanford CA USA; ^3^ Department of Pediatrics, Lucile Packard Children's Hospital Stanford, Stanford University School of Medicine Stanford University Stanford CA USA; ^4^ Department of Radiology, New York‐Presbyterian Morgan Stanley Children's Hospital Columbia University Irving Medical Center New York NY USA

**Keywords:** High‐risk neuroblastoma; DXA; Sarcopenia; Areal bone mineral density; Autologous stem cell transplantation; *cis*‐Retinoic acid

## Abstract

**Background:**

Survival from paediatric high‐risk neuroblastoma (HR‐NBL) has increased, but *cis*‐retinoic acid (*cis*‐RA), the cornerstone of HR‐NBL therapy, can cause osteoporosis and premature physeal closure and is a potential threat to skeletal structure in HR‐NBL survivors. Sarcopenia is associated with increased morbidity in survivors of paediatric malignancies. Low muscle mass may be associated with poor prognosis in HR‐NBL patients but has not been studied in these survivors. The study objective was to assess bone density, body composition and muscle strength in HR‐NBL survivors compared with controls.

**Methods:**

This prospective cross‐sectional study assessed areal bone mineral density (aBMD) of the whole body, lumbar spine, total hip, femoral neck, distal 1/3 and ultradistal radius and body composition (muscle and fat mass) using dual‐energy X‐ray absorptiometry (DXA) and lower leg muscle strength using a dynamometer. Measures expressed as sex‐specific standard deviation scores (Z‐scores) included aBMD (adjusted for height Z‐score), bone mineral apparent density (BMAD), leg lean mass (adjusted for leg length), whole‐body fat mass index (FMI) and ankle dorsiflexion peak torque adjusted for leg length (strength‐Z). Muscle‐specific force was assessed as strength relative to leg lean mass. Outcomes were compared between HR‐NBL survivors and controls using Student's *t‐*test or Mann–Whitney *U* test. Linear regression models examined correlations between DXA and dynamometer outcomes.

**Results:**

We enrolled 20 survivors of HR‐NBL treated with *cis*‐RA [13 male; mean age: 12.4 ± 1.6 years; median (range) age at therapy initiation: 2.6 (0.3–9.1) years] and 20 age‐, sex‐ and race‐matched controls. Height‐Z was significantly lower in HR‐NBL survivors compared with controls (−1.73 ± 1.38 vs. 0.34 ± 1.12, *P* < 0.001). Areal BMD‐Z, BMAD‐Z, FMI‐Z, visceral adipose tissue and subcutaneous adipose tissue were not significantly different in HR‐NBL survivors compared with controls. Compared with controls, HR‐NBL survivors had lower leg lean mass‐Z (−1.46 ± 1.35 vs. − 0.17 ± 0.84, *P* < 0.001) and strength‐Z (−1.13 ± 0.86 vs. − 0.15 ± 0.71, *P* < 0.001). Muscle‐specific force was lower in HR‐NBL survivors compared with controls (*P* < 0.05).

**Conclusions:**

Bone mineral density and adiposity are not severely impacted in HR‐NBL survivors with growth failure, but significant sarcopenia persists years after treatment. Future studies are needed to determine if sarcopenia improves with muscle‐specific interventions in this population of cancer survivors.

## Introduction

Therapeutic advances have transformed the prognosis for high‐risk neuroblastoma (HR‐NBL) into a curable disease, and the current 5‐year event‐free survival now approaches 50%.^1^ These survival improvements arise from intensive multimodal approaches including high‐dose chemotherapy, surgery, tandem autologous stem cell transplant (ASCT), radiation therapy, immunotherapy and biologic agents such as *cis*‐retinoic acid (*cis*‐RA).^2^

Long‐term survivors of HR‐NBL exhibit a severe burden of late effects.^3^ The combination of young age at diagnosis and aggressive multimodal treatment, even in the absence of total body irradiation (TBI), leads to marked growth failure and short stature.^4^ We recently reported differences in physeal structure between HR‐NBL survivors and healthy controls, suggesting poor growth plate architecture as the underlying cause for abnormal growth.^5^ However, body composition abnormalities have not been well characterized in HR‐NBL survivors. Metabolic syndrome at rates higher than healthy controls has been identified in survivors of neuroblastoma, but not specifically HR‐NBL, who received abdominal radiation.^6^ To date, few studies have examined bone mineral deficits and body composition aberrations in survivors after HR‐NBL therapy, including immunotherapy and the biologic agent *cis*‐RA.^7^

*cis*‐RA is a vitamin A derivative with multiple effects, including osteoporosis and premature physeal plate closure.^8^, ^9^ Guinea pigs treated with high doses of vitamin A demonstrate physeal closure in the tibia and femur mediated by retinoic acid receptors, as well as weight loss and poor growth.^10^ Histological examination of cells treated with retinoic acid reveals decreased physeal plate thickness and disintegration of the growth plate.^11^, ^12^ A case series identified three patients who developed premature physeal growth arrest after isotretinoin exposure, with greater incidence of bony abnormalities in patients diagnosed between ages 5 and 10 than in those diagnosed before the age of 5 and those diagnosed after the age of 10.^13^ These studies suggest that treatment of HR‐NBL with *cis*‐RA contributes to bony abnormalities in younger age, which is associated with substantial skeletal morbidity in adulthood, including limb shortening, deformity, fractures and osteonecrosis.^14^

Survivors of HR‐NBL have numerous risk factors for abnormal body composition, such as nutritional deficiencies and endocrine abnormalities as a consequence of chemotherapy, ASCT and radiation.^6^ Sarcopenia is the progressive loss of muscle mass and function caused by ageing, disease and inactivity; it is associated with physical disability and premature mortality in survivors of ASCT.^15^, ^16^, ^17^ Sarcopenia has been demonstrated in other populations of cancer survivors.^17^, ^18^ Most studies describing body composition abnormalities in survivors of childhood cancer include a small number of patients and an insufficiently robust control population necessary to characterize body composition relative to age, sex, race, maturation and body size. As a result, abnormalities in body composition in long‐term survivors of HR‐NBL have not been well characterized.

The objective of this study was to assess areal bone mineral density (aBMD) and body composition using dual‐energy X‐ray absorptiometry (DXA) in HR‐NBL survivors treated with *cis*‐RA as part of neuroblastoma therapy. DXA is a method of comprehensive bone and body composition assessment in children.^19^ We have previously used DXA to demonstrate body composition abnormalities in long‐term survivors of paediatric haematopoietic stem cell transplantation and acute lymphoblastic leukaemia (ALL).^20^ We apply these techniques to a population of long‐term survivors of HR‐NBL to examine aBMD, lean mass, fat mass, visceral adiposity and subcutaneous adiposity. Further, we examine muscle‐specific strength for functional assessment. We hypothesized that HR‐NBL survivors would have muscle deficits compared with age‐, sex‐ and race‐matched controls. Our primary outcome was muscle mass and strength. Secondary outcome was aBMD in order to better understand the functional muscle‐bone unit and the impact of muscle on bone.

## Methods

### Study sample

This was a prospective study of 20 survivors with history of HR‐NBL diagnosed and treated at the Children's Hospital of Philadelphia (CHOP) and 20 healthy age‐, sex‐ and race‐matched controls. All survivors were recruited from CHOP oncology and endocrinology clinics. Inclusion criteria included (1) diagnosis with HR‐NBL per ANBL00B1 enrolment, (2) treatment with *cis‐*RA as part of HR‐NBL therapy, (3) age 6–16 years at time of enrolment and (4) in complete remission for at least 2 years from completion of therapy. Exclusion criteria included (1) treatment with bisphosphonates, (2) estimated glomerular filtration rate < 60 mL/min/1.73 m^2^,^21^ (3) full skeletal maturity determined by response to pubertal development questionnaire and/or fusion of growth plates on bone age evaluation, (4) active malignancy, (5) pregnancy or (6) greater than 1‐year post‐menarche. Study participants and their parent(s) were asked to categorize the participant's race according to the National Institute of Health categories. Sex‐, race‐ and age‐ (within 1 year) matched controls were recruited from practices in the community consistent with our prior studies and excluded based on (1) history of cancer; (2) hepatic, renal, thyroid, neuromuscular or joint disease; (3) skeletal maturity; (4) malabsorption syndromes; (5) medications impacting growth or BMD; ( 6) use of oral retinoids for acne; or (7) pregnancy.^22^

The study was approved by the CHOP Institutional Review Board, complied with Health Insurance Portability and Accountability Act (HIPAA) guidelines, and was performed in accordance with the Declaration of Helsinki. Informed parental consent and assent were obtained for all study participants.

### Anthropometry and physical maturity

Height was measured with a stadiometer (Holtain, Crymych, UK), and weight with a digital scale (Scale Tronix, White Plains, NY). Sitting height was measured with a stadiometer (Holtain, Crymych, UK) using a standardized stool. Sitting height was defined as the difference between the height of the participant seated on the stool and the height of the stool. Sitting height index was defined as the ratio of sitting height to standing height. Leg length was defined as the difference between standing height and sitting height. Tanner pubertal stage was determined by a paediatric endocrinologist (SMM) in HR‐NBL participants and by validated self‐assessment questionnaire in the control participants.^23^ For calculation of dorsiflexion Z‐scores, tibia length was measured from the distal margin of the medial malleolus to the proximal border of the medial tibial condyle.

### HR‐NBL disease and treatment characteristics

Participants were treated as per the Children's Oncology Group High Risk Neuroblastoma Consortium protocols CCG‐3891, CHP‐594, ANBL0532, ANBL0931, ANBL09P1, ANBL0032 and/or ANBL1221. Medical charts were reviewed for date of diagnosis, neuroblastoma primary site, metastatic burden, detailed HR‐NBL treatment regimen including cumulative dose of *cis‐*RA, number of ASCT, treatment response, presence or absence of relapse, additional relapse treatment regimens and time since therapy completion. Other history recorded included diagnosed and treated endocrine late effects such as thyroid hormone, growth hormone and gonadal hormone replacements and all current medications.

As previously described,^5^ survivors with growth hormone deficiency were diagnosed using conventional assessments, which included growth failure, abnormal growth velocity for age, laboratory testing and formal stimulation testing of the growth axis.

The cumulative dose of *cis*‐RA for each participant was calculated as the sum of total milligram dose (mg/m^2^ body surface area, multiplied by the participant's body surface area) for all cycles of therapy, as previously described.^5^

### DXA

DXA scans were acquired using a Hologic Discovery bone densitometer (Hologic, Bedford, MA). Whole‐body and non‐dominant forearm scans were acquired in the array mode. Posterior anterior lumbar spine (L1–L4) and left hip scans were acquired in the fast array mode. Scans were analysed using software versions 12.3 and 12.4 to generate areal BMD (g/cm^3^) and BMC (g). Spine phantoms were scanned daily, and total body phantoms were scanned weekly. The precision error for aBMD and BMC was <1% for the spine and <2.5% for the total body phantoms, respectively.

DXA measures of leg lean mass, appendicular lean mass and total body lean mass (kg) (excluding bone mass) were obtained from the total body scan as an index of skeletal muscle in order to assess the relationship between muscle and bone, as previously described.^20^ We have demonstrated that total body lean mass underestimated muscle deficits in childhood cancer survivors due to altered body proportions (longer legs relative to height) following spine radiation.^24^

DXA whole‐body fat mass (FM, kg) (excluding head) was obtained as a measure of overall body adiposity. To interpret fat mass relative to height, fat mass index (FMI) was calculated [FM/(height)^2^]. DXA abdominal visceral adipose tissue (VAT) and subcutaneous adipose tissue (SAT) area (cm^2^) were quantified in a 5‐cm region at the L_4_ level (Hologic APEX 3.1 software) with VAT coefficient of variation reported at 2.3%.^25^

Three HR‐NBL survivors were excluded from analysis of hip scans (total hip and femoral neck), and one HR‐NBL survivor was excluded from analysis of lumbar spine due to the presence of indwelling hardware (secondary to treatment for scoliosis and/or slipped capital femoral epiphysis). These four HR‐NBL survivors were also excluded from analysis of TBLH aBMD.

### Measurement of muscle torque

Muscle torque was assessed by isometric ankle dynamometry using the Biodex Multi‐Joint System 3 Pro dynamometer (Biodex Medical Systems, Inc., Shirley, NY). Muscle torque is the gold standard for measuring muscle contractile force.^26^ For this system, high intra‐rater (0.97–0.99) and inter‐rater (0.93–0.96) intraclass correlation coefficients have been reported.^27^ Peak isometric torque (ft‐lbs) was measured in triplicate at 20° plantarflexion, and the highest value was recorded for dorsiflexion of the ankle. The test–retest reproducibility in our laboratory for this measure (coefficient of variation) is 4.3%.^26^

### Physical activity

Physical activity was assessed in the HR‐NBL survivors and matched control participants using a questionnaire that captured over 30 sports and play activities, summarized as h/week.^28^

### Fracture data

HR‐NBL and control participant fracture summary was captured based on retrospective self‐reported data. Low‐impact fractures were defined as fractures occurring after falls from standing height or lower, without major trauma.

### Laboratory studies

Laboratory studies were performed in HR‐NBL survivors only. IGF‐1 levels (mg/dL) were measured by ELISA (R&D Systems, Minneapolis, MN), and Z‐scores were calculated based on the manufacturer's normal ranges for age and Tanner stage.

### Statistical analysis

Stata 16.0 (Stata Corp., College Station, TX) was used for all statistical analyses. Age‐ and sex‐specific Z‐scores for height, weight and body mass index were calculated using National Center for Health Statistics data.^29^ Sitting height and leg length Z‐scores were calculated using reference ranges from the National Health and Nutrition Examination Survey (NHANES).^30^, ^31^ Bone and body composition measures are highly correlated with height. Therefore, adjustment for short stature was performed for interpretation of bone and body composition measures.^32^, ^33^ Age‐, sex‐ and race‐specific Z‐scores were calculated for DXA aBMD, BMC and leg lean mass using national reference data from the Bone Mineral Density in Childhood Study (BMDCS).^34^ Height‐for‐age‐Z‐specific Z‐scores were calculated for aBMD and BMC; leg length‐for‐age‐specific Z‐scores were calculated for leg lean mass.^32^ Age‐, sex‐ and race‐specific lumbar spine bone mineral apparent density (BMAD) Z‐scores (BMAD‐Z) were developed using BMDCS reference data.^35^ Sex‐specific FMI Z‐scores (FMI‐Z**)**, appendicular lean mass index Z‐scores and total body lean mass index Z‐scores were developed using NHANES reference data.^36^

Dorsiflexion peak torque Z‐scores (strength‐Z) were calculated based on age, sex, race and tibia length.^37^ We examined muscle torque relative to muscle size as a measure of muscle quality^26^, ^38^ by evaluating the differences in strength‐Z relative to HR‐NBL survivorship status (HR‐NBL survivors compared with controls) with a linear regression, adjusted for leg lean mass‐Z. Reference data were not available for VAT and SAT area. Therefore, differences in VAT and SAT area relative to HR‐NBL survivorship status (HR‐NBL survivors compared with controls) were evaluated using linear regression, adjusted for the covariates age, sex, FMI‐Z and sitting height‐Z. Pearson's correlation was used to calculate correlation coefficients for DXA BMD, fat mass and lean mass outcomes and IGF‐1‐Z or 25(OH)D level.

Categorical data were compared between HR‐NBL survivors and controls using Pearson's chi‐square test or Fisher's exact test. Distributions of all continuous variables were examined for normality. Depending upon data normality, group differences in continuous variables were assessed using Student's *t‐*test or Mann–Whitney *U* test. Per cent of subjects with low Z‐scores in each group was inspected. A *P‐*value of <0.05 was considered statistically significant, and two‐sided tests of hypotheses were used throughout.

## Results

### Participant disease and treatment characteristics

Forty participants (20 survivors of HR‐NBL and 20 healthy matched controls; 13 males and 7 females per group) were recruited during a 17‐month period. We prospectively enrolled 20 (44%) of the total 45 eligible patients contacted. Participant characteristics did not differ between enrolled and eligible patients who declined study participation. The group characteristics are summarized in *Table* 1, as previously described by our group.^5^ HR‐NBL survivors and control participants were matched for age (HR‐NBL participant mean age: 12.45 years, range: 9.46–16.03; control participant mean age: 12.18 years, range: 10.36–14.11). The oldest survivor had a younger control for comparison as we were unable to identify an age‐matched control with open growth plates. None of the participants in the study had history of skeletal trauma. HR‐NBL survivors and control participants did not differ in the number of low‐impact fractures or total fractures (*Table* 1).

**Table 1:** Characteristics in high‐risk neuroblastoma (HR‐NBL) and matched‐control participants

	HR‐NBL (*n* = 20)	Matched controls^a^ (*n* = 20)	*P‐*value
Age at study enrolment, years	12.4 ± 1.6	12.2 ± 1.1	‐‐
Sex, male, *n* (%)	13 (65%)	13 (65%)	‐‐
Race, black, *n* (%)	4 (20%)	4 (20%)	‐‐
Pubertal status, *n* (%)			
Tanner Stage 1	10 (50%)	6 (30%)	0.07
Tanner Stages 2–3	9 (45%)	8 (40%)	
Tanner Stages 4–5	1 (5%)	6 (30%)	
Weight (kg)	35.8 ± 9.9	46.5 ± 12.6, 44.0^d^ (32.3–86.4)	<0.005
Weight Z‐score	−1.30 ± 1.43	0.34 ± 0.96	<0.001
Sitting height (cm)	73.0 ± 6.1	79.7 ± 5.4	<0.001
Sitting height Z‐score	−1.38 ± 1.26	0.42 ± 1.04	<0.001
Sitting height index Z‐score^b^	0.68 ± 1.22	0.43 ± 0.86	0.45
Height (cm)	139.7 ± 12.0	153.5 ± 10.1	<0.001
Height Z‐score	−1.73 ± 1.38	0.34 ± 1.12	<0.001
BMI (kg/m^2^)	18.0 ± 2.4	19.5 ± 3.6, 18.8^d^ (16.3–30.8)	0.13
BMI Z‐score	−0.25 ± 0.93	0.30 ± 0.90	0.09
Tibia length (cm)	32.3 ± 0.8	35.7 ± 0.6	<0.005
Leg length for age Z‐score	−1.77 ± 1.31	0.10 ± 1.23	<0.001
Physical activity level^c^	2.6 ± 0.7	2.4 ± 0.6	0.47
Fracture history (any)	6 (30%)	8 (40%)	0.52
Low‐impact fracture history	1 (5%)	1 (5%)	‐‐
TBLH aBMD height‐adjusted Z‐score	0.61 ± 1.17	0.58 ± 0.91	0.95
Lumbar spine aBMD height‐adjusted Z‐score	0.57 ± 1.30	−0.08 ± 0.90	0.08
Lumbar spine BMAD Z‐score	0.36 ± 1.49, 0.70^d^ (−3.47–2.06)	−0.05 ± 0.97	0.12
Total hip aBMD height‐adjusted Z‐score	0.04 ± 1.18	−0.07 ± 0.96	0.74
Femoral neck aBMD height‐adjusted Z‐score	−0.77 ± 1.11	−0.25 ± 0.90	0.13
Distal 1/3 radius aBMD height‐adjusted Z‐score	0.02 ± 1.07	0.22 ± 0.87, −0.05^d^ (−0.73–2.81)	0.96
Ultradistal radius aBMD height‐adjusted Z‐score	0.28 ± 1.71	−0.18 ± 1.07	0.32
Total body lean mass index Z‐score	−1.21 ± 1.10	−0.30 ± 0.83	<0.01
Appendicular lean mass index Z‐score	−1.38 ± 1.27	−0.49 ± 0.91	*<*0.05
Leg lean mass adjusted for leg length Z‐score	−1.46 ± 1.35	−0.17 ± 0.84	<0.001
Dorsiflexion peak torque Z‐score	−1.13 ± 0.86	−0.15 ± 0.71	<0.001

Values are *n* (%) or mean ± standard deviation.

BMI, body mass index; TBLH, total body less head; BMAD, bone mineral apparent density.

^a^ Controls were sex, race and age matched (±1 year) to high‐risk neuroblastoma participants.

^b^ Sitting height index was defined as the ratio of sitting height to standing height.

^c^ Physical activity level was quantified using the Physical Activity Questionnaire for Older Children (PAQ‐C) or Adolescents (PAQ‐A), where applicable.^28^

^d^ Data were skewed so values are also reported as median (range).

There were no significant differences in demographic characteristics of the HR‐NBL and control participants (*Table* 1). HR‐NBL was associated with delayed pubertal maturation. As shown in *Table* 1, mean height Z‐score for HR‐NBL survivors was lower compared with that of controls (−1.73 ± 1.38 vs. 0.34 ± 1.12, *P* < 0.001). Mean sitting height Z‐score was lower for HR‐NBL survivors compared with that of controls (−1.38 ± 1.26 vs. 0.42 ± 1.04, *P* < 0.001). However, sitting height index Z‐score did not differ between HR‐NBL survivors and controls. The mean leg length Z‐score was lower for HR‐NBL survivors compared with that of controls (−1.77 ± 1.31 vs. 0.10 ± 1.23, *P* < 0.001).

*Table* 2 summarizes HR‐NBL disease and treatment characteristics. Seventeen HR‐NBL survivors (85%) were diagnosed with an endocrinopathy and treated with hormone replacement therapy. Twelve survivors (60%) received growth hormone for deficiency.

**Table 2:** Disease and treatment characteristics in high‐risk neuroblastoma participants

**Characteristics**	
Age at study enrolment, years	12.2 (9.5–15.8)
Age at diagnosis, years	2.8 (0.3–9.1)
Time since diagnosis, years	9.0 (3.0–15.5)
Total body irradiation	7 (35%)
*cis*‐Retinoic acid	20 (100%)
Cumulative dose (mg)	8450 (5040–27,580)
IGF‐1 Z‐score^a^	1.15 (−2.10–5.70)
Endocrine abnormalities	
Hypothyroidism on treatment	10 (50%)
Growth hormone deficient	15 (75%)
On growth hormone therapy at visit	12 (60%)
Response to growth hormone therapy	6 (50%)

Values are *n* (%) or median (range).

^a^ IGF‐1 level adjusted for age and Tanner stage.

Fifteen HR‐NBL survivors received six cycles of *cis*‐RA (*Table* 2). Three survivors received slightly different courses (5.5, 7 and 9 cycles), and two received extended *cis*‐RA treatment (14 and 17 cycles), due to relapse and refractory disease. Eight HR‐NBL survivors (40%) received TBI as part of ASCT conditioning treatment regimen (*Table* 2).

### aBMD measurements

**Figure 1:**
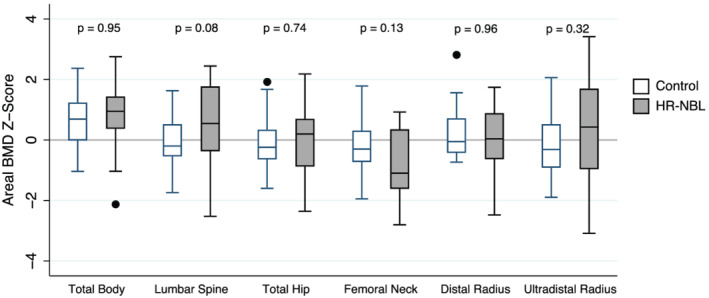
Height‐for‐age‐Z‐adjusted areal BMD (aBMD) Z‐scores for high‐risk neuroblastoma (HR‐NBL) and matched‐control participants.

Height‐for‐age‐Z‐adjusted aBMD Z‐scores at all six sites examined (total body less head, lumbar spine, total hip, femoral neck, distal 1/3 radius and ultradistal radius) did not differ between HR‐NBL participants and controls (*Table* 1and *Figure* 1). One HR‐NBL participant had low for age (Z‐score ≤ −2.0) HAZ‐adjusted aBMD‐Z at all six sites tested; this participant did not receive TBI, was not growth hormone deficient and had delayed pubertal development with no history of hormone replacement therapy. HAZ‐adjusted aBMD Z‐scores less than −1.0 at the femoral neck were more common in HR‐NBL participants compared with controls (*n* = 9 vs. *n* = 4). Lumbar spine BMAD‐Z did not differ between HR‐NBL participants and controls (*Table* 1).

### IGF‐1 levels

IGF‐1 Z‐scores were low for age and pubertal status in four HR‐NBL survivors. Of those with low IGF‐1 Z‐scores, one male was growth hormone deficient and had been previously treated with growth hormone, one male was growth hormone deficient with no history of growth hormone treatment, and one female was growth hormone deficient with no history of growth hormone treatment. One female did not have prior diagnosis of growth hormone deficiency and had not been treated with growth hormone. Greater total body less head aBMD‐Z (*R* = 0.72, *P* < 0.001), lumbar spine aBMD‐Z (*R* = 0.59, *P* = 0.01), total hip aBMD‐Z (*R* = 0.54, *P* = 0.047), ultradistal radius aBMD‐Z (R = 0.59, P = 0.01) and leg lean mass‐Z (*R* = 0.53, *P* = 0.03) were associated with greater IGF‐1‐Z.

### Adiposity

HR‐NBL participants had FMI‐Z comparable with matched controls (−0.18 ± 0.64 vs. − 0.11 ± 0.89, *P* = 0.76). DXA VAT and SAT areas (cm^2^) were also comparable between HR‐NBL participants and controls [VAT area median (range): 33.8 (16.7–86.3) vs. 35.0 (11.2–95.8), *P* = 0.89; SAT area 99.6 (26.3–278.3) vs. 124.5 (28.4–537.7), *P* = 0.79]. The lack of difference in VAT and SAT areas between HR‐NBL participants and matched controls persisted in a linear regression analysis with covariates age, sex, FMI‐Z and sitting height‐Z.

### Lean body mass

**Figure 2:**
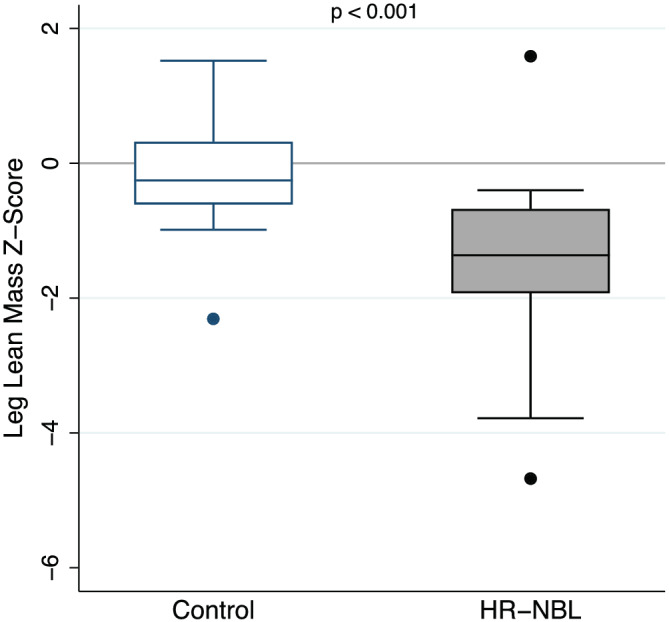
Leg length‐adjusted lean mass Z‐scores for high‐risk neuroblastoma (HR‐NBL) and matched‐control participants. Figures were created using Stata 16.0 (Stata Corp., College Station, TX) and Adobe Photoshop (Adobe, San Jose, CA).

Leg length‐adjusted leg lean mass Z‐score was significantly lower in HR‐NBL participants compared with controls (−1.46 ± 1.35 vs. − 0.17 ± 0.84, *P* < 0.001) (*Table* 1 and *Figure* 2). Leg lean mass Z‐scores less than −2.0 were more common in HR‐NBL participants compared with controls (*n* = 4 vs. *n* = 1). Similarly, leg lean mass Z‐scores less than −1.0 were more common in HR‐NBL participants compared with controls (*n* = 11 vs. n = 1). Leg lean mass Z‐scores were highly correlated with total body lean mass index Z‐scores and appendicular lean mass index Z‐scores (*R* = 0.9130, *P* < 0.001 and *R* = 0.9323, *P* < 0.001, respectively).

### Muscle strength and quality measurements

Strength‐Z was lower in HR‐NBL participants compared with controls (−1.13 ± 0.86 vs. − 0.15 ± 0.71, *P* < 0.001) (*Table* 1). Furthermore, assessment of muscle‐specific force through multivariate regression models demonstrated that the negative association between HR‐NBL status and strength‐Z remained significant when adjusted for leg length‐adjusted lean mass Z‐score (*Table* 3).

**Table 3:** Differences in dorsiflexion Z‐score outcome relative to high‐risk neuroblastoma (HR‐NBL) survivor status, with and without adjustment for leg lean mass Z‐score

	*β* (95% CI) *P*‐value	
	HR‐NBL relative to control participant status	HR‐NBL relative to control participant status, adjusted for leg lean mass Z‐score
Difference in dorsiflexion Z‐score	−0.98 (−1.40 to −0.46) *P* < 0.001	−0.60 (−1.14 to −0.06) *P* = 0.03

CI, confidence interval.

## Discussion

This is the first study to examine bone density, body composition and muscle strength outcomes in long‐term survivors of HR‐NBL. In this contemporary cohort of survivors of HR‐NBL treated with *cis‐*RA, we did not find bone density deficits after accounting for their short stature. However, we found significant deficits in lean mass with low muscle‐specific strength relative to age‐, sex‐ and race‐matched healthy controls. The persistent low muscle mass and strength (sarcopenia) are present in these survivors, years after completion of HR‐NBL therapy.

There is a paucity of studies assessing sarcopenia in HR‐NBL. Kawakubo et al.^39^ performed a limited retrospective study to assess the relationship between low muscle mass and HR‐NBL outcome. Based on CT‐derived psoas muscle area in 13 HR‐NBL patients, those who maintained muscle quantity over the course of HR‐NBL treatment had longer progression‐free survival than patients who lost muscle mass over their treatment course. Patients who relapsed or died had lower psoas muscle area compared with patients with progression‐free survival. These results highlight the importance of low muscle mass as a potential marker of poor prognosis in patients diagnosed with HR‐NBL. However, this study by Kawakubo et al. was limited by absence of functional muscle assessment in HR‐NBL patients. In our study, we performed functional assessment using dynamometry, demonstrating low muscle mass and low muscle‐specific strength in HR‐NBL survivors, meeting the definition of sarcopenia.^15^, ^16^ Muscle was qualitatively different in HR‐NBL survivors compared with controls, given that differences were present even after adjustment for lower leg lean mass.

There are several potential causes for sarcopenia in HR‐NBL survivors. The effects of intensive treatment, including ASCT, cause disruption to the bone marrow milieu and functional muscle–bone unit. Therapy for HR‐NBL remains a highly intense, multi‐agent treatment regimen, and no singular treatment primarily contributes to the sarcopenia in HR‐NBL survivors. Recipients of bone marrow transplant who receive similar chemotherapy and radiation regimens demonstrate comparable lasting deficits.^17^, ^24^ In addition, low physical activity and nutritional deficiency as an effect of treatment may also contribute to sarcopenia. Our findings are in line with the presence of sarcopenia in other recipients of ASCT such as Hodgkin or non‐Hodgkin lymphoma.^17^ The identification of sarcopenic obesity in these survivors was also associated with increased mortality risk at 1 year and at 5 years after ASCT compared with patients with normal body composition.^17^ In a cohort of 19 HR‐NBL survivors, Vatanen et al.^40^ identified increased frailty (defined as having three or more of the following: low muscle mass, low energy expenditure, slow running and weakness) among HR‐NBL participants compared with age‐ and sex‐matched controls. This study also identified significantly shorter telomere length and higher serum levels of high sensitivity C‐reactive protein in HR‐NBL participants compared with controls, suggestive of premature ageing. Despite comparable levels of physical activity years after treatment in our cohort of HR‐NBL survivors compared with controls, levels of physical activity may have been significantly lower during and shortly after completion of HR‐NBL treatment. Taken together, the concerning findings of sarcopenia and premature ageing in survivors of HR‐NBL suggest the need to intervene and treat muscle deficits as a serious late effect of intense HR‐NBL treatment.

The findings of lean mass and muscle strength deficits are in line with our previous work in long‐term survivors of allogeneic haematopoietic cell transplant.^24^ These transplant recipients exhibited sarcopenic obesity, with greater trunk VAT area and whole‐body fat mass compared with age‐, sex‐ and race‐matched controls.^24^ In contrast, HR‐NBL survivors demonstrated sarcopenia with no significant differences in adipose measures compared with controls, potentially due to less adipocyte differentiation from multipotent mesenchymal stem cells related to differences in cancer treatment regimen. In recipients of ASCT, sarcopenia alone was associated with an increased mortality risk at 1 year and at 5 years after ASCT compared with patients with normal body composition,^17^ underlining the importance of identifying sarcopenia in HR‐NBL survivors who received ASCT.

We identified lower height Z‐score, sitting height Z‐score and leg length Z‐score in HR‐NBL survivors compared with matched controls with preserved sitting height index Z‐score, demonstrating growth failure with proportionately shorter bones. These anthropometric results indicate a defect in overall growth, potentially due to direct effects of *cis‐*RA on the growth plate and secondary effects of growth hormone deficiency, gonadal hormone deficiency and nutritional deficits. The high prevalence of endocrine dysfunction identified in this study highlight the importance of routine endocrinology follow‐up and provocative hormone testing for these high‐risk survivors.

No differences in height‐for‐age‐Z‐adjusted aBMD or BMAD Z‐scores were identified between HR‐NBL and control participants. These results are consistent with results from Utriainen et al., who found that aBMD adjusted for bone size did not differ between HR‐NBL survivors and age‐ and sex‐matched controls.^41^ Despite significant short stature even in the absence of TBI, our results suggest relative preservation of BMD. Further, the lack of increased fractures in this cohort of HR‐NBL survivors is reassuring for preserved bone health. The correlation between greater IGF‐1 Z‐score in HR‐NBL survivors and greater leg lean mass Z‐score and aBMD Z‐score at the total body less head, lumbar spine, total hip and ultradistal radius is likely driven by the underlying growth hormone treatment received by HR‐NBL survivors with growth hormone deficiency, given that the growth hormone–IGF‐1 axis drives the development of muscle and bone.

In contrast to our findings of preserved BMD in survivors of HR‐NBL, we have previously shown that survivors of ALL have low total hip and femoral neck aBMD Z‐scores compared with national reference data.^20^ Moreover, long‐term survivors of paediatric allogeneic haematopoietic cell transplant had lower trabecular volumetric BMD compared with reference data.^18^ Compared with these two populations, the preservation of BMD in long‐term survivors of HR‐NBL may be due to protective effects of ASCT preserving the bone marrow trophic environment.

The primary limitations of our study are the cross‐sectional design, modest sample size, lack of muscle biopsies and lack of laboratory measurements in the matched controls. Absence of longitudinal evaluation prohibits assessment of changes in DXA or dynamometry outcomes over time. The extended duration since completion of treatment prohibits examining the immediate impact of *cis*‐RA, chemotherapy and ASCT on bone and body composition. Finally, the study did not use objective measures of physical activity, such as use of accelerometers.

However, this study has many important strengths. We present the first prospective study to evaluate lean mass in HR‐NBL survivors using DXA and muscle function using dynamometry, demonstrating low muscle strength after adjusting for low lean mass—consistent with both low muscle mass and abnormal quality. We provide a thorough assessment of bone outcomes at multiple DXA sites following multimodal therapy for HR‐NBL. Although BMD deficits have been identified in survivors of other paediatric malignancies, this cohort of HR‐NBL survivors exhibited appropriate bone health for their degree of short stature. We also recruited a comparison group of age‐, sex‐ and race‐matched healthy controls.

In conclusion, the combination of markedly decreased leg lean mass and decreased muscle‐specific strength underline the substantial burden of long‐term sarcopenia, with the need for earlier interventions to target muscle development in HR‐NBL patients. Rather than targeting interventions years after completion of HR‐NBL treatment, we anticipate that muscle gains may be most impactful if muscle is targeted in early treatment stages. Rigorous proactive exercise interventions, such as those employed for ALL,^42^, ^43^ may have a role in the routine prophylactic treatment of HR‐NBL. Future longitudinal studies are essential to determine the mechanism of sarcopenia and long‐term complications secondary to sarcopenia and to identify strategies that not only promote normal muscle accrual but also improve muscle‐specific strength in the growing number of HR‐NBL survivors.

## Funding

This study was supported by grants from the Institute for Translational Medicine and Therapeutics of the Perelman School of Medicine at the University of Pennsylvania (Guo), the National Institutes of Health/National Cancer Institute (CA166177 [Mostoufi‐Moab]) and St. Baldrick's Foundation (Mostoufi‐Moab).

Financial Benefits to Author.

The authors will not benefit financially from publication of this material.

## Conflict of interest

All authors report no conflict of interest.

## Author contributions

MG, SMM and DJ designed the study; MG and SMM collected, assembled, analysed and interpreted the data and wrote the paper; DJ, BSZ, JL, MBL, AK and CPH analysed and interpreted the data; MG, SMM, DJ, BSZ, JL, MBL, AK and CPH reviewed and critiqued the manuscript and contributed to revisions. All authors approved the final manuscript.

## References

[1] George RE , Li S , Medeiros‐Nancarrow C , Neuberg D , Marcus K , Shamberger RC , et al. High‐risk neuroblastoma treated with tandem autologous peripheral‐blood stem cell‐supported transplantation: long‐term survival update. J Clin Oncol 2006;24:2891–2896. 16782928 10.1200/JCO.2006.05.6986

[2] Matthay KK , Villablanca JG , Seeger RC , Stram DO , Harris RE , Ramsay NK , et al. Treatment of high‐risk neuroblastoma with intensive chemotherapy, radiotherapy, autologous bone marrow transplantation, and 13‐cis‐retinoic acid. Children's Cancer Group. N Engl J Med 1999;341:1165–1173. 10519894 10.1056/NEJM199910143411601

[3] Hobbie WL , Moshang T , Carlson CA , Goldmuntz E , Sacks N , Goldfarb SB , et al. Late effects in survivors of tandem peripheral blood stem cell transplant for high‐risk neuroblastoma. Pediatr Blood Cancer 2008;51:679–683. 18623215 10.1002/pbc.21683 PMC2888471

[4] Cohen LE , Gordon JH , Popovsky EY , Gunawardene S , Duffey‐Lind E , Lehmann LE , et al. Late effects in children treated with intensive multimodal therapy for high‐risk neuroblastoma: high incidence of endocrine and growth problems. Bone Marrow Transplant 2014;49:502–508. 24442245 10.1038/bmt.2013.218

[5] Delgado J , Jaramillo D , Chauvin NA , Guo M , Stratton MS , Sweeney HE , et al. Evaluating growth failure with diffusion tensor imaging in pediatric survivors of high‐risk neuroblastoma treated with high‐dose cis‐retinoic acid. Pediatr Radiol 2019;49:1056–1065. 31055614 10.1007/s00247-019-04409-1 PMC6599475

[6] van Waas M , Neggers SJ , Raat H , van Rij CM , Pieters R , van den Heuvel‐Eibrink MM . Abdominal radiotherapy: a major determinant of metabolic syndrome in nephroblastoma and neuroblastoma survivors. PLoS One 2012;7:e52237. 23251703 10.1371/journal.pone.0052237 PMC3522621

[7] Robison LL , Armstrong GT , Boice JD , Chow EJ , Davies SM , Donaldson SS , et al. The childhood cancer survivor study: a National Cancer Institute‐supported resource for outcome and intervention research. J Clin Oncol 2009;27:2308–2318. 19364948 10.1200/JCO.2009.22.3339 PMC2677920

[8] Kaplan GHaettich B . Rheumatological symptoms due to retinoids. Baillieres Clin Rheumatol 1991;5:77–97. 2070429 10.1016/s0950-3579(05)80297-3

[9] Rothenberg AB , Berdon WE , Woodard JC , Cowles RA . Hypervitaminosis A‐induced premature closure of epiphyses (physeal obliteration) in humans and calves (hyena disease): a historical review of the human and veterinary literature. Pediatr Radiol 2007;37:1264–1267. 17909784 10.1007/s00247-007-0604-0

[10] Standeven AM , Davies PJ , Chandraratna RA , Mader DR , Johnson AT , Thomazy VA . Retinoid‐induced epiphyseal plate closure in guinea pigs. Fundam Appl Toxicol 1996;34:91–98. 8937896 10.1006/faat.1996.0179

[11] Horton WE , Yamada Y , Hassell JR . Retinoic acid rapidly reduces cartilage matrix synthesis by altering gene transcription in chondrocytes. Dev Biol 1987;123:508–516. 3653521 10.1016/0012-1606(87)90409-x

[12] Benya PD , Padilla SR . Modulation of the rabbit chondrocyte phenotype by retinoic acid terminates type II collagen synthesis without inducing type I collagen: the modulated phenotype differs from that produced by subculture. Dev Biol 1986;118:296–305. 3770304 10.1016/0012-1606(86)90096-5

[13] Duvalyan A , Cha A , Goodarzian F , Arkader A , Villablanca JG , Marachelian A . Premature epiphyseal growth plate arrest after isotretinoin therapy for high‐risk neuroblastoma: a case series and review of the literature. Pediatr Blood Cancer 2020;67:e28236. 32386124 10.1002/pbc.28236

[14] Rayar MS , Nayiager T , Webber CE , Barr RD , Athale UH . Predictors of bony morbidity in children with acute lymphoblastic leukemia. Pediatr Blood Cancer 2012;59:77–82. 22190454 10.1002/pbc.24040

[15] Cruz‐Jentoft AJ , Sayer AA . Sarcopenia. Lancet 2019;393:2636–2646. 31171417 10.1016/S0140-6736(19)31138-9

[16] Pratesi A , Tarantini F , Di Bari M . Skeletal muscle: an endocrine organ. Clin Cases Miner Bone Metab 2013;10:11–14. 23858303 10.11138/ccmbm/2013.10.1.011 PMC3710002

[17] Armenian SH , Iukuridze A , Teh JB , Mascarenhas K , Herrera A , McCune JS , et al. Abnormal body composition is a predictor of adverse outcomes after autologous haematopoietic cell transplantation. J Cachexia Sarcopenia Muscle 2020;11:962–972. 32212263 10.1002/jcsm.12570 PMC7432567

[18] Mostoufi‐Moab S , Ginsberg JP , Bunin N , Zemel BS , Shults J , Thayu M , et al. Body composition abnormalities in long‐term survivors of pediatric hematopoietic stem cell transplantation. J Pediatr 2012;160:122–128. 21839468 10.1016/j.jpeds.2011.06.041 PMC3218257

[19] Simoni P , Guglielmi R , Aparisi Gomez MP . Imaging of body composition in children. Quant Imaging Med Surg 2020;10:1661–1671. 32742959 10.21037/qims.2020.04.06 PMC7378095

[20] Mostoufi‐Moab S , Kelly A , Mitchell JA , Baker J , Zemel BS , Brodsky J , et al. Changes in pediatric DXA measures of musculoskeletal outcomes and correlation with quantitative CT following treatment of acute lymphoblastic leukemia. Bone 2018;112:128–135. 29679731 10.1016/j.bone.2018.04.012 PMC5970089

[21] Schwartz GJ , Work DF . Measurement and estimation of GFR in children and adolescents. Clin J Am Soc Nephrol 2009;4:1832–1843. 19820136 10.2215/CJN.01640309

[22] Leonard MB , Elmi A , Mostoufi‐Moab S , Shults J , Burnham JM , Thayu M , et al. Effects of sex, race, and puberty on cortical bone and the functional muscle bone unit in children, adolescents, and young adults. J Clin Endocrinol Metab 2010;95:1681–1689. 20157194 10.1210/jc.2009-1913 PMC2853999

[23] Tanner J , Whitehouse R , Marshall W , Healy M , Goldstein H . Assessment of skeletal maturity and prediction of adult height (TW3) method. London: WB Saunders; 2001.

[24] Mostoufi‐Moab S , Magland J , Isaacoff EJ , Sun W , Rajapakse CS , Zemel B , et al. Adverse fat depots and marrow adiposity are associated with skeletal deficits and insulin resistance in long‐term survivors of pediatric hematopoietic stem cell Transplantation. J Bone Miner Res 2015;30:1657–1666. 25801428 10.1002/jbmr.2512 PMC4540662

[25] Micklesfield LK , Goedecke JH , Punyanitya M , Wilson KE , Kelly TL . Dual‐energy X‐ray performs as well as clinical computed tomography for the measurement of visceral fat. Obesity (Silver Spring) 2012;20:1109–1114. 22240726 10.1038/oby.2011.367 PMC3343346

[26] Lee DY , Wetzsteon RJ , Zemel BS , Shults J , Organ JM , Foster BJ , et al. Muscle torque relative to cross‐sectional area and the functional muscle‐bone unit in children and adolescents with chronic disease. J Bone Miner Res 2015;30:575–583. 25264231 10.1002/jbmr.2375 PMC4532328

[27] Leggin BG , Neuman RM , Iannotti JP , Williams GR , Thompson EC . Intrarater and interrater reliability of three isometric dynamometers in assessing shoulder strength. J Shoulder Elbow Surg 1996;5:18–24. 8919438 10.1016/s1058-2746(96)80026-7

[28] Kowalski KC , Crocker PRE , Donen RM . The Physical Activity Questionnaire for Older Children (PAQ‐C) and Adolescents (PAQ‐A) Manual. Saskatoon, SK, Canada: University of Saskatchewan; 2004.

[29] Ogden CL , Kuczmarski RJ , Flegal KM , Mei Z , Guo S , Wei R , et al. Centers for Disease Control and Prevention 2000 growth charts for the United States: improvements to the 1977 National Center for Health Statistics version. Pediatrics 2002;109:45–60. 11773541 10.1542/peds.109.1.45

[30] Hawkes CP , Mostoufi‐Moab S , McCormack SE , Grimberg A , Zemel BS . Sitting height to standing height ratio reference charts for children in the United States. J Pediatr 2020;226:221–227.e15. 10.1016/j.jpeds.2020.06.051 PMC9030919 32579888

[31] Hawkes CP , Mostoufi‐Moab S , McCormack SE , Grimberg A , Zemel BS . Leg length and sitting height reference data and charts for children in the United States. Data Brief 2020;32:106131. 32904356 10.1016/j.dib.2020.106131 PMC7452688

[32] Zemel BS , Leonard MB , Kelly A , Lappe JM , Gilsanz V , Oberfield S , et al. Height adjustment in assessing dual energy x‐ray absorptiometry measurements of bone mass and density in children. J Clin Endocrinol Metab 2010;95:1265–1273. 20103654 10.1210/jc.2009-2057 PMC2841534

[33] Crabtree NJ , Arabi A , Bachrach LK , Fewtrell M , El‐Hajj Fuleihan G , Kecskemethy HH , et al. Dual‐energy X‐ray absorptiometry interpretation and reporting in children and adolescents: the revised 2013 ISCD Pediatric Official Positions. J Clin Densitom 2014;17:225–242. 24690232 10.1016/j.jocd.2014.01.003

[34] Zemel BS , Kalkwarf HJ , Gilsanz V , Lappe JM , Oberfield S , Shepherd JA , et al. Revised reference curves for bone mineral content and areal bone mineral density according to age and sex for black and non‐black children: results of the bone mineral density in childhood study. J Clin Endocrinol Metab 2011;96:3160–3169. 21917867 10.1210/jc.2011-1111 PMC3200252

[35] Kindler JM , Lappe JM , Gilsanz V , Oberfield S , Shepherd JA , Kelly A , et al. Lumbar spine bone mineral apparent density in children: results from the bone mineral density in childhood study. J Clin Endocrinol Metab 2019;104:1283–1292. 30265344 10.1210/jc.2018-01693 PMC6397436

[36] Weber DR , Moore RH , Leonard MB , Zemel BS . Fat and lean BMI reference curves in children and adolescents and their utility in identifying excess adiposity compared with BMI and percentage body fat. Am J Clin Nutr 2013;98:49–56. 23697708 10.3945/ajcn.112.053611 PMC3683820

[37] Leonard MB , Zemel BS , Wrotniak BH , Klieger SB , Shults J , Stallings VA , et al. Tibia and radius bone geometry and volumetric density in obese compared to non‐obese adolescents. Bone 2015;73:69–76. 25497572 10.1016/j.bone.2014.12.002 PMC4540475

[38] Avitabile CM , Leonard MB , Zemel BS , Brodsky JL , Lee D , Dodds K , et al. Lean mass deficits, vitamin D status and exercise capacity in children and young adults after Fontan palliation. Heart 2014;100:1702–1707. 24973081 10.1136/heartjnl-2014-305723 PMC4386691

[39] Kawakubo N , Kinoshita Y , Souzaki R , Koga Y , Oba U , Ohga S , et al. The influence of sarcopenia on high‐risk neuroblastoma. J Surg Res 2019;236:101–105. 30694741 10.1016/j.jss.2018.10.048

[40] Vatanen A , Hou M , Huang T , Soder O , Jahnukainen T , Kurimo M , et al. Clinical and biological markers of premature aging after autologous SCT in childhood cancer. Bone Marrow Transplant 2017;52:600–605. 28067869 10.1038/bmt.2016.334

[41] Utriainen P , Vatanen A , Toiviainen‐Salo S , Saarinen‐Pihkala U , Makitie O , Jahnukainen K . Skeletal outcome in long‐term survivors of childhood high‐risk neuroblastoma treated with high‐dose therapy and autologous stem cell rescue. Bone Marrow Transplant 2017;52:711–716. 28067882 10.1038/bmt.2016.345

[42] Tanner L , Sencer S , Hooke MC . The Stoplight program: a proactive physical therapy intervention for children with acute lymphoblastic leukemia. J Pediatr Oncol Nurs 2017;34:347–357. 28459187 10.1177/1043454217698093

[43] Manchola‐Gonzalez JD , Bagur‐Calafat C , Girabent‐Farres M , Serra‐Grima JR , Perez RA , Garnacho‐Castano MV , et al. Effects of a home‐exercise programme in childhood survivors of acute lymphoblastic leukaemia on physical fitness and physical functioning: results of a randomised clinical trial. Support Care Cancer 2020;28:3171–3178. 31707503 10.1007/s00520-019-05131-2

[44] von Haehling S , Morley JE , Coats AJS , Anker SD . Ethical guidelines for publishing in the Journal of Cachexia, Sarcopenia and Muscle: update 2019. J Cachexia Sarcopenia Muscle 2019;10:1143–1145. 31661195 10.1002/jcsm.12501 PMC6818444

